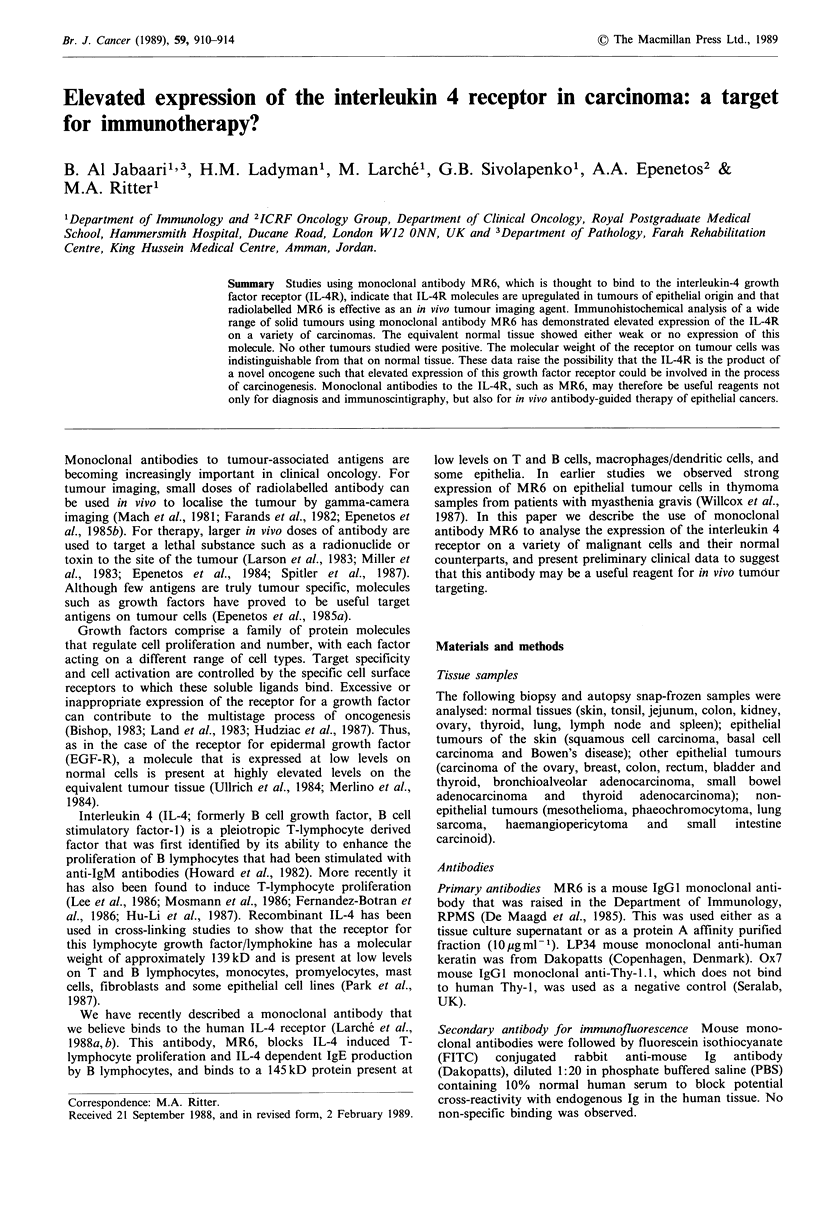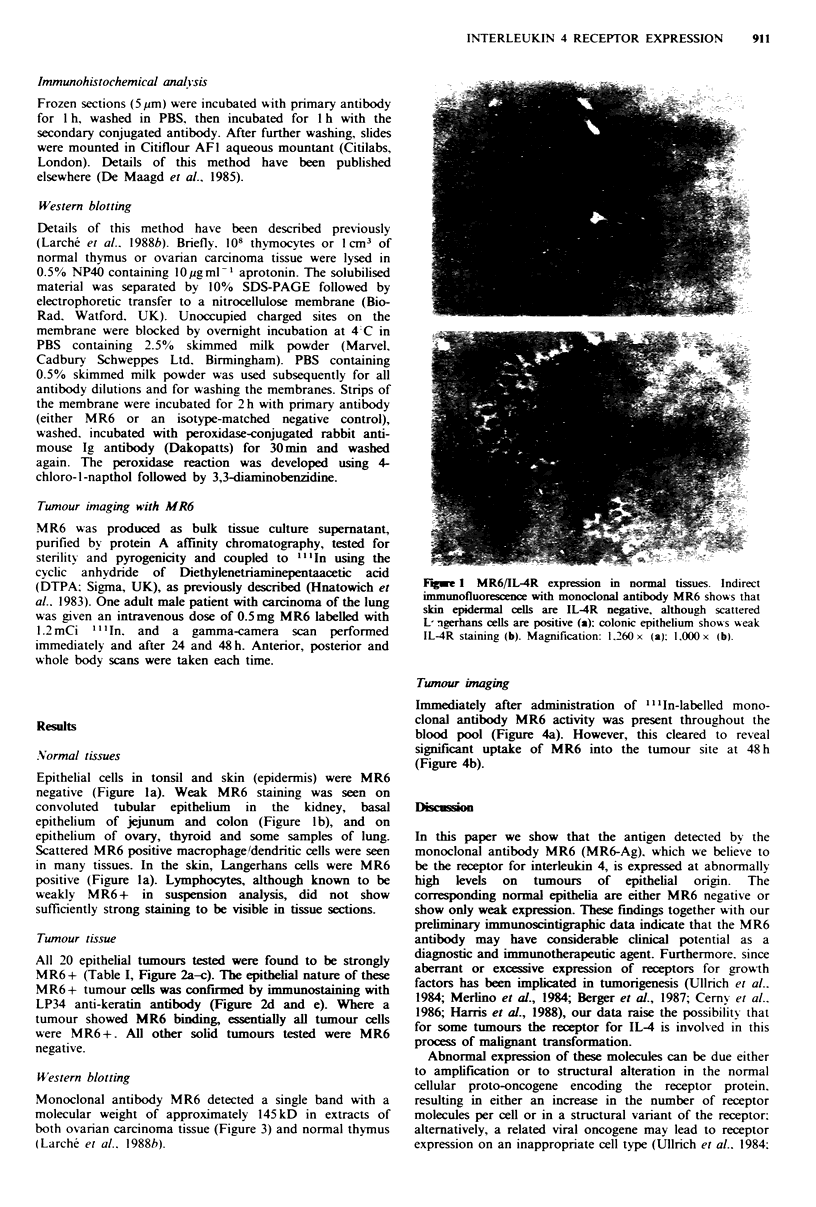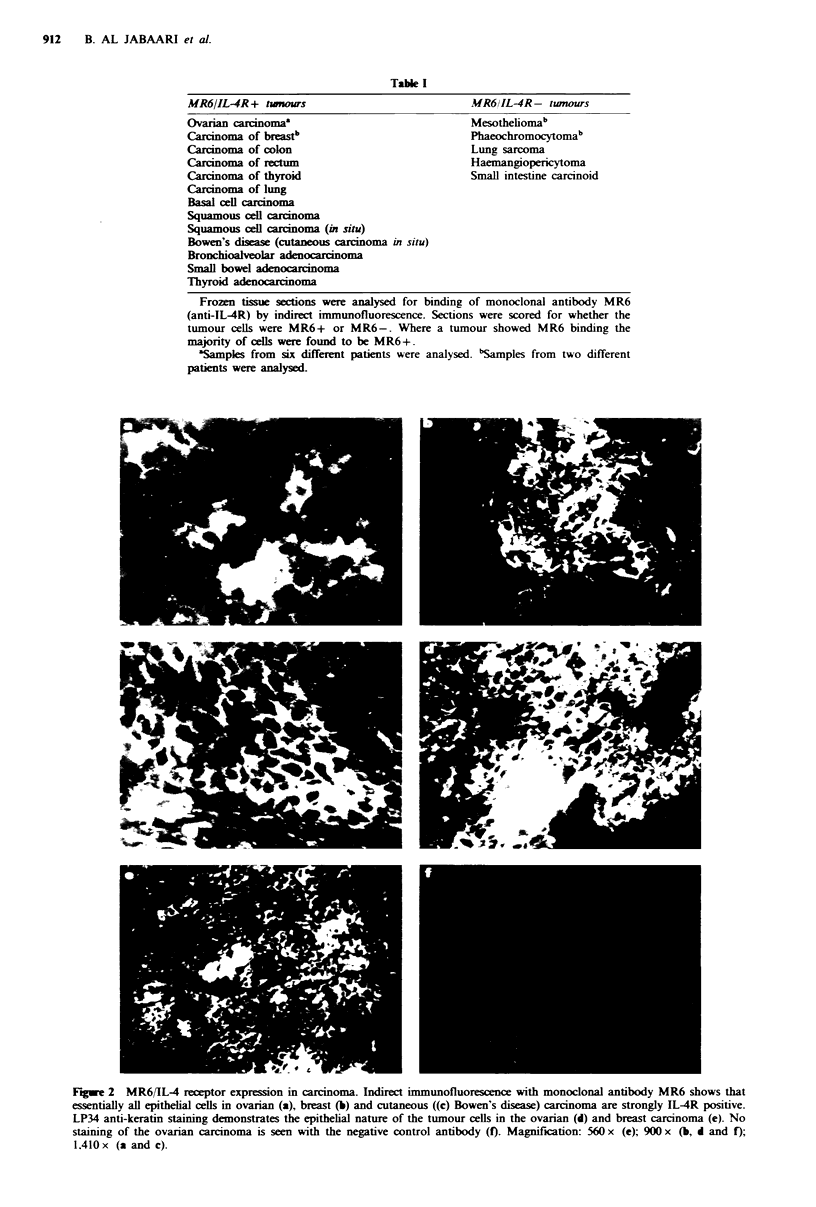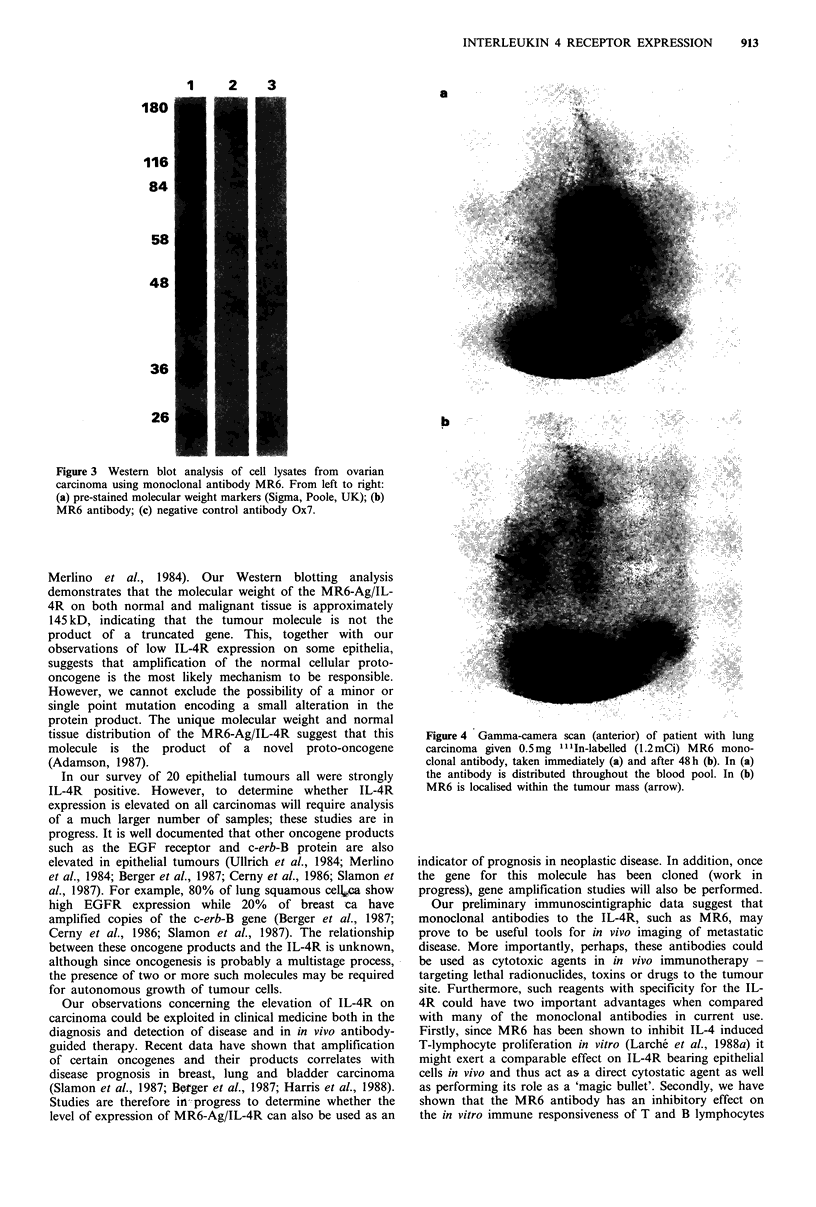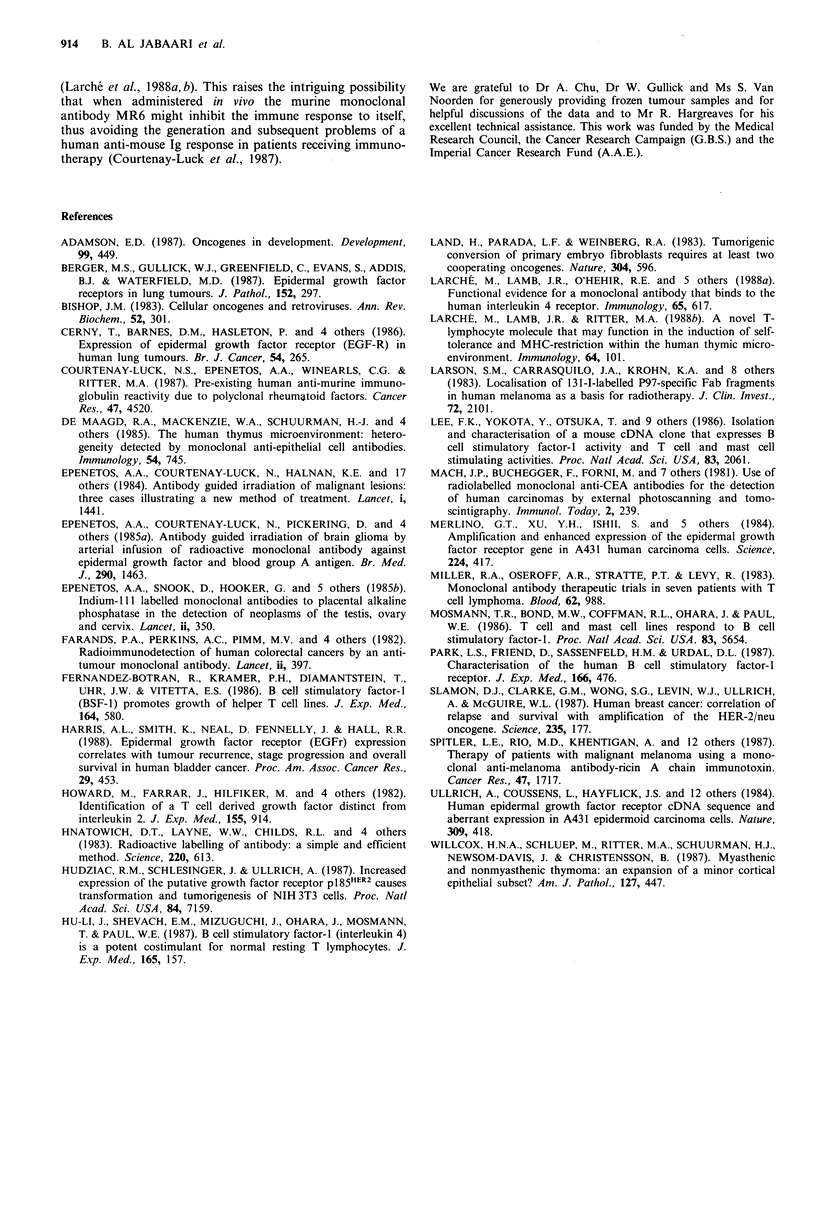# Elevated expression of the interleukin 4 receptor in carcinoma: a target for immunotherapy?

**DOI:** 10.1038/bjc.1989.192

**Published:** 1989-06

**Authors:** B. Al Jabaari, H. M. Ladyman, M. LarchÃ©, G. B. Sivolapenko, A. A. Epenetos, M. A. Ritter

**Affiliations:** Department of Immunology, Royal Postgraduate Medical School, Hammersmith Hospital, London.

## Abstract

**Images:**


*Br. J. Cancer* (1989), **59**, 910–914 © The Macmillan Press Ltd., 1989

### Elevated expression of the interleukin 4 receptor in carcinoma: a target for immunotherapy?

B. Al Jabaari[1,3], H.M. Ladyman[1], M. Larché[1], G.B. Sivolapenko[1], A.A. Epenetos[2] & M.A. Ritter[1]

[1]*Department of Immunology and* [2]*ICRF Oncology Group, Department of Clinical Oncology, Royal Postgraduate Medical School, Hammersmith Hospital, Ducane Road, London W12 0NN, UK and* [3]*Department of Pathology, Farah Rehabilitation Centre, King Hussein Medical Centre, Amman, Jordan.*

**Summary** Studies using monoclonal antibody MR6, which is thought to bind to the interleukin-4 growth factor receptor (IL-4R), indicate that IL-4R molecules are upregulated in tumours of epithelial origin and that radiolabelled MR6 is effective as an *in vivo* tumour imaging agent. Immunohistochemical analysis of a wide range of solid tumours using monoclonal antibody MR6 has demonstrated elevated expression of the IL-4R on a variety of carcinomas. The equivalent normal tissue showed either weak or no expression of this molecule. No other tumours studied were positive. The molecular weight of the receptor on tumour cells was indistinguishable from that on normal tissue. These data raise the possibility that the IL-4R is the product of a novel oncogene such that elevated expression of this growth factor receptor could be involved in the process of carcinogenesis. Monoclonal antibodies to the IL-4R, such as MR6, may therefore be useful reagents not only for diagnosis and immunoscintigraphy, but also for *in vivo* antibody-guided therapy of epithelial cancers.

Monoclonal antibodies to tumour-associated antigens are becoming increasingly important in clinical oncology. For tumour imaging, small doses of radiolabelled antibody can be used *in vivo* to localise the tumour by gamma-camera imaging (Mach *et al.*, 1981; Farands *et al.*, 1982; Epenetos *et al.*, 1985*b*). For therapy, larger *in vivo* doses of antibody are used to target a lethal substance such as a radionuclide or toxin to the site of the tumour (Larson *et al.*, 1983; Miller *et al.*, 1983; Epenetos *et al.*, 1984; Spitler *et al.*, 1987). Although few antigens are truly tumour specific, molecules such as growth factors have proved to be useful target antigens on tumour cells (Epenetos *et al.*, 1985*a*).

Growth factors comprise a family of protein molecules that regulate cell proliferation and number, with each factor acting on a different range of cell types. Target specificity and cell activation are controlled by the specific cell surface receptors to which these soluble ligands bind. Excessive or inappropriate expression of the receptor for a growth factor can contribute to the multistage process of oncogenesis (Bishop, 1983; Land *et al.*, 1983; Hudziac *et al.*, 1987). Thus, as in the case of the receptor for epidermal growth factor (EGF-R), a molecule that is expressed at low levels on normal cells is present at highly elevated levels on the equivalent tumour tissue (Ullrich *et al.*, 1984; Merlino *et al.*, 1984).

Interleukin 4 (IL-4; formerly B cell growth factor, B cell stimulatory factor-1) is a pleiotropic T-lymphocyte derived factor that was first identified by its ability to enhance the proliferation of B lymphocytes that had been stimulated with anti-IgM antibodies (Howard *et al.*, 1982). More recently it has also been found to induce T-lymphocyte proliferation (Lee *et al.*, 1986; Mosmann *et al.*, 1986; Fernandez-Botran *et al.*, 1986; Hu-Li *et al.*, 1987). Recombinant IL-4 has been used in cross-linking studies to show that the receptor for this lymphocyte growth factor/lymphokine has a molecular weight of approximately 139 kD and is present at low levels on T and B lymphocytes, monocytes, promyelocytes, mast cells, fibroblasts and some epithelial cell lines (Park *et al.*, 1987).

We have recently described a monoclonal antibody that we believe binds to the human IL-4 receptor (Larché *et al.*, 1988*a*, *b*). This antibody, MR6, blocks IL-4 induced T-lymphocyte proliferation and IL-4 dependent IgE production by B lymphocytes, and binds to a 145 kD protein present at low levels on T and B cells, macrophages/dendritic cells, and some epithelia. In earlier studies we observed strong expression of MR6 on epithelial tumour cells in thymoma samples from patients with myasthenia gravis (Willcox *et al.*, 1987). In this paper we describe the use of monoclonal antibody MR6 to analyse the expression of the interleukin 4 receptor on a variety of malignant cells and their normal counterparts, and present preliminary clinical data to suggest that this antibody may be a useful reagent for *in vivo* tumour targeting.

Correspondence: M.A. Ritter.
Received 21 September 1988, and in revised form, 2 February 1989.

### Materials and methods

### *Tissue samples*

The following biopsy and autopsy snap-frozen samples were analysed: normal tissues (skin, tonsil, jejunum, colon, kidney, ovary, thyroid, lung, lymph node and spleen); epithelial tumours of the skin (squamous cell carcinoma, basal cell carcinoma and Bowen's disease); other epithelial tumours (carcinoma of the ovary, breast, colon, rectum, bladder and thyroid, bronchioalveolar adenocarcinoma, small bowel adenocarcinoma and thyroid adenocarcinoma); non-epithelial tumours (mesothelioma, phaeochromocytoma, lung sarcoma, haemangiopericytoma and small intestine carcinoid).

### *Antibodies*

*Primary antibodies* MR6 is a mouse IgG1 monoclonal antibody that was raised in the Department of Immunology, RPMS (De Maagd *et al.*, 1985). This was used either as a tissue culture supernatant or as a protein A affinity purified fraction ($10\,\mu g\,ml^{-1}$). LP34 mouse monoclonal anti-human keratin was from Dakopatts (Copenhagen, Denmark). Ox7 mouse IgG1 monoclonal anti-Thy-1.1, which does not bind to human Thy-1, was used as a negative control (Seralab, UK).

*Secondary antibody for immunofluorescence* Mouse monoclonal antibodies were followed by fluorescein isothiocyanate (FITC) conjugated rabbit anti-mouse Ig antibody (Dakopatts), diluted 1:20 in phosphate buffered saline (PBS) containing 10% normal human serum to block potential cross-reactivity with endogenous Ig in the human tissue. No non-specific binding was observed.

*Immunohistochemical analysis*

Frozen sections (5 μm) were incubated with primary antibody for 1 h, washed in PBS, then incubated for 1 h with the secondary conjugated antibody. After further washing, slides were mounted in Citiflour AF1 aqueous mountant (Citilabs, London). Details of this method have been published elsewhere (De Maagd *et al.*, 1985).

*Western blotting*

Details of this method have been described previously (Larché *et al.*, 1988*b*). Briefly, $10^8$ thymocytes or 1 $cm^3$ of normal thymus or ovarian carcinoma tissue were lysed in 0.5% NP40 containing 10 $\mu g\,ml^{-1}$ aprotonin. The solubilised material was separated by 10% SDS-PAGE followed by electrophoretic transfer to a nitrocellulose membrane (Bio-Rad, Watford, UK). Unoccupied charged sites on the membrane were blocked by overnight incubation at 4°C in PBS containing 2.5% skimmed milk powder (Marvel, Cadbury Schweppes Ltd, Birmingham). PBS containing 0.5% skimmed milk powder was used subsequently for all antibody dilutions and for washing the membranes. Strips of the membrane were incubated for 2 h with primary antibody (either MR6 or an isotype-matched negative control), washed, incubated with peroxidase-conjugated rabbit anti-mouse Ig antibody (Dakopatts) for 30 min and washed again. The peroxidase reaction was developed using 4-chloro-1-napthol followed by 3,3-diaminobenzidine.

*Tumour imaging with MR6*

MR6 was produced as bulk tissue culture supernatant, purified by protein A affinity chromatography, tested for sterility and pyrogenicity and coupled to $^{111}In$ using the cyclic anhydride of Diethylenetriaminepentaacetic acid (DTPA; Sigma, UK), as previously described (Hnatowich *et al.*, 1983). One adult male patient with carcinoma of the lung was given an intravenous dose of 0.5 mg MR6 labelled with 1.2 mCi $^{111}In$, and a gamma-camera scan performed immediately and after 24 and 48 h. Anterior, posterior and whole body scans were taken each time.

### Results

*Normal tissues*

Epithelial cells in tonsil and skin (epidermis) were MR6 negative (Figure 1a). Weak MR6 staining was seen on convoluted tubular epithelium in the kidney, basal epithelium of jejunum and colon (Figure 1b), and on epithelium of ovary, thyroid and some samples of lung. Scattered MR6 positive macrophage/dendritic cells were seen in many tissues. In the skin, Langerhans cells were MR6 positive (Figure 1a). Lymphocytes, although known to be weakly MR6+ in suspension analysis, did not show sufficiently strong staining to be visible in tissue sections.

*Tumour tissue*

All 20 epithelial tumours tested were found to be strongly MR6+ (Table I, Figure 2a–c). The epithelial nature of these MR6+ tumour cells was confirmed by immunostaining with LP34 anti-keratin antibody (Figure 2d and e). Where a tumour showed MR6 binding, essentially all tumour cells were MR6+. All other solid tumours tested were MR6 negative.

*Western blotting*

Monoclonal antibody MR6 detected a single band with a molecular weight of approximately 145 kD in extracts of both ovarian carcinoma tissue (Figure 3) and normal thymus (Larché *et al.*, 1988*b*).

**Figure 1** MR6/IL-4R expression in normal tissues. Indirect immunofluorescence with monoclonal antibody MR6 shows that skin epidermal cells are IL-4R negative, although scattered L'ngerhans cells are positive (**a**); colonic epithelium shows weak IL-4R staining (**b**). Magnification: 1,260 × (**a**); 1,000 × (**b**).

*Tumour imaging*

Immediately after administration of $^{111}In$-labelled monoclonal antibody MR6 activity was present throughout the blood pool (Figure 4a). However, this cleared to reveal significant uptake of MR6 into the tumour site at 48 h (Figure 4b).

### Discussion

In this paper we show that the antigen detected by the monoclonal antibody MR6 (MR6-Ag), which we believe to be the receptor for interleukin 4, is expressed at abnormally high levels on tumours of epithelial origin. The corresponding normal epithelia are either MR6 negative or show only weak expression. These findings together with our preliminary immunoscintigraphic data indicate that the MR6 antibody may have considerable clinical potential as a diagnostic and immunotherapeutic agent. Furthermore, since aberrant or excessive expression of receptors for growth factors has been implicated in tumorigenesis (Ullrich *et al.*, 1984; Merlino *et al.*, 1984; Berger *et al.*, 1987; Cerny *et al.*, 1986; Harris *et al.*, 1988), our data raise the possibility that for some tumours the receptor for IL-4 is involved in this process of malignant transformation.

Abnormal expression of these molecules can be due either to amplification or to structural alteration in the normal cellular proto-oncogene encoding the receptor protein, resulting in either an increase in the number of receptor molecules per cell or in a structural variant of the receptor; alternatively, a related viral oncogene may lead to receptor expression on an inappropriate cell type (Ullrich *et al.*, 1984;

Table I

| *MR6/IL-4R+ tumours* | *MR6/IL-4R− tumours* |
|---|---|
| Ovarian carcinoma[a] | Mesothelioma[b] |
| Carcinoma of breast[b] | Phaeochromocytoma[b] |
| Carcinoma of colon | Lung sarcoma |
| Carcinoma of rectum | Haemangiopericytoma |
| Carcinoma of thyroid | Small intestine carcinoid |
| Carcinoma of lung | |
| Basal cell carcinoma | |
| Squamous cell carcinoma | |
| Squamous cell carcinoma (*in situ*) | |
| Bowen's disease (cutaneous carcinoma *in situ*) | |
| Bronchioalveolar adenocarcinoma | |
| Small bowel adenocarcinoma | |
| Thyroid adenocarcinoma | |

Frozen tissue sections were analysed for binding of monoclonal antibody MR6 (anti-IL-4R) by indirect immunofluorescence. Sections were scored for whether the tumour cells were MR6+ or MR6−. Where a tumour showed MR6 binding the majority of cells were found to be MR6+.

[a]Samples from six different patients were analysed. [b]Samples from two different patients were analysed.

**Figure 2** MR6/IL-4 receptor expression in carcinoma. Indirect immunofluorescence with monoclonal antibody MR6 shows that essentially all epithelial cells in ovarian (**a**), breast (**b**) and cutaneous ((**c**) Bowen's disease) carcinoma are strongly IL-4R positive. LP34 anti-keratin staining demonstrates the epithelial nature of the tumour cells in the ovarian (**d**) and breast carcinoma (**e**). No staining of the ovarian carcinoma is seen with the negative control antibody (**f**). Magnification: 560 × (**e**); 900 × (**b**, **d** and **f**); 1,410 × (**a** and **c**).

**Figure 3** Western blot analysis of cell lysates from ovarian carcinoma using monoclonal antibody MR6. From left to right: (**a**) pre-stained molecular weight markers (Sigma, Poole, UK); (**b**) MR6 antibody; (**c**) negative control antibody Ox7.

Merlino *et al.*, 1984). Our Western blotting analysis demonstrates that the molecular weight of the MR6-Ag/IL-4R on both normal and malignant tissue is approximately 145 kD, indicating that the tumour molecule is not the product of a truncated gene. This, together with our observations of low IL-4R expression on some epithelia, suggests that amplification of the normal cellular proto-oncogene is the most likely mechanism to be responsible. However, we cannot exclude the possibility of a minor or single point mutation encoding a small alteration in the protein product. The unique molecular weight and normal tissue distribution of the MR6-Ag/IL-4R suggest that this molecule is the product of a novel proto-oncogene (Adamson, 1987).

In our survey of 20 epithelial tumours all were strongly IL-4R positive. However, to determine whether IL-4R expression is elevated on all carcinomas will require analysis of a much larger number of samples; these studies are in progress. It is well documented that other oncogene products such as the EGF receptor and c-*erb*-B protein are also elevated in epithelial tumours (Ullrich *et al.*, 1984; Merlino *et al.*, 1984; Berger *et al.*, 1987; Cerny *et al.*, 1986; Slamon *et al.*, 1987). For example, 80% of lung squamous cell ca show high EGFR expression while 20% of breast ca have amplified copies of the c-*erb*-B gene (Berger *et al.*, 1987; Cerny *et al.*, 1986; Slamon *et al.*, 1987). The relationship between these oncogene products and the IL-4R is unknown, although since oncogenesis is probably a multistage process, the presence of two or more such molecules may be required for autonomous growth of tumour cells.

Our observations concerning the elevation of IL-4R on carcinoma could be exploited in clinical medicine both in the diagnosis and detection of disease and in *in vivo* antibody-guided therapy. Recent data have shown that amplification of certain oncogenes and their products correlates with disease prognosis in breast, lung and bladder carcinoma (Slamon *et al.*, 1987; Berger *et al.*, 1987; Harris *et al.*, 1988). Studies are therefore in progress to determine whether the level of expression of MR6-Ag/IL-4R can also be used as an

**Figure 4** Gamma-camera scan (anterior) of patient with lung carcinoma given 0.5 mg $^{111}$In-labelled (1.2 mCi) MR6 monoclonal antibody, taken immediately (**a**) and after 48 h (**b**). In (**a**) the antibody is distributed throughout the blood pool. In (**b**) MR6 is localised within the tumour mass (arrow).

indicator of prognosis in neoplastic disease. In addition, once the gene for this molecule has been cloned (work in progress), gene amplification studies will also be performed.

Our preliminary immunoscintigraphic data suggest that monoclonal antibodies to the IL-4R, such as MR6, may prove to be useful tools for *in vivo* imaging of metastatic disease. More importantly, perhaps, these antibodies could be used as cytotoxic agents in *in vivo* immunotherapy – targeting lethal radionuclides, toxins or drugs to the tumour site. Furthermore, such reagents with specificity for the IL-4R could have two important advantages when compared with many of the monoclonal antibodies in current use. Firstly, since MR6 has been shown to inhibit IL-4 induced T-lymphocyte proliferation *in vitro* (Larché *et al.*, 1988*a*) it might exert a comparable effect on IL-4R bearing epithelial cells *in vivo* and thus act as a direct cytostatic agent as well as performing its role as a 'magic bullet'. Secondly, we have shown that the MR6 antibody has an inhibitory effect on the *in vitro* immune responsiveness of T and B lymphocytes

(Larché *et al.*, 1988*a*, *b*). This raises the intriguing possibility that when administered *in vivo* the murine monoclonal antibody MR6 might inhibit the immune response to itself, thus avoiding the generation and subsequent problems of a human anti-mouse Ig response in patients receiving immunotherapy (Courtenay-Luck *et al.*, 1987).

We are grateful to Dr A. Chu, Dr W. Gullick and Ms S. Van Noorden for generously providing frozen tumour samples and for helpful discussions of the data and to Mr R. Hargreaves for his excellent technical assistance. This work was funded by the Medical Research Council, the Cancer Research Campaign (G.B.S.) and the Imperial Cancer Research Fund (A.A.E.).

### References

ADAMSON, E.D. (1987). Oncogenes in development. *Development*, **99**, 449.

BERGER, M.S., GULLICK, W.J., GREENFIELD, C., EVANS, S., ADDIS, B.J. & WATERFIELD, M.D. (1987). Epidermal growth factor receptors in lung tumours. *J. Pathol.*, **152**, 297.

BISHOP, J.M. (1983). Cellular oncogenes and retroviruses. *Ann. Rev. Biochem.*, **52**, 301.

CERNY, T., BARNES, D.M., HASLETON, P. and 4 others (1986). Expression of epidermal growth factor receptor (EGF-R) in human lung tumours. *Br. J. Cancer*, **54**, 265.

COURTENAY-LUCK, N.S., EPENETOS, A.A., WINEARLS, C.G. & RITTER, M.A. (1987). Pre-existing human anti-murine immunoglobulin reactivity due to polyclonal rheumatoid factors. *Cancer Res.*, **47**, 4520.

DE MAAGD, R.A., MACKENZIE, W.A., SCHUURMAN, H.-J. and 4 others (1985). The human thymus microenvironment: heterogeneity detected by monoclonal anti-epithelial cell antibodies. *Immunology*, **54**, 745.

EPENETOS, A.A., COURTENAY-LUCK, N., HALNAN, K.E. and 17 others (1984). Antibody guided irradiation of malignant lesions: three cases illustrating a new method of treatment. *Lancet*, **i**, 1441.

EPENETOS, A.A., COURTENAY-LUCK, N., PICKERING, D. and 4 others (1985*a*). Antibody guided irradiation of brain glioma by arterial infusion of radioactive monoclonal antibody against epidermal growth factor and blood group A antigen. *Br. Med. J.*, **290**, 1463.

EPENETOS, A.A., SNOOK, D., HOOKER, G. and 5 others (1985*b*). Indium-111 labelled monoclonal antibodies to placental alkaline phosphatase in the detection of neoplasms of the testis, ovary and cervix. *Lancet*, **ii**, 350.

FARANDS, P.A., PERKINS, A.C., PIMM, M.V. and 4 others (1982). Radioimmunodetection of human colorectal cancers by an antitumour monoclonal antibody. *Lancet*, **ii**, 397.

FERNANDEZ-BOTRAN, R., KRAMER, P.H., DIAMANTSTEIN, T., UHR, J.W. & VITETTA, E.S. (1986). B cell stimulatory factor-1 (BSF-1) promotes growth of helper T cell lines. *J. Exp. Med.*, **164**, 580.

HARRIS, A.L., SMITH, K., NEAL, D. FENNELLY, J. & HALL, R.R. (1988). Epidermal growth factor receptor (EGFr) expression correlates with tumour recurrence, stage progression and overall survival in human bladder cancer. *Proc. Am. Assoc. Cancer Res.*, **29**, 453.

HOWARD, M., FARRAR, J., HILFIKER, M. and 4 others (1982). Identification of a T cell derived growth factor distinct from interleukin 2. *J. Exp. Med.*, **155**, 914.

HNATOWICH, D.T., LAYNE, W.W., CHILDS, R.L. and 4 others (1983). Radioactive labelling of antibody: a simple and efficient method. *Science*, **220**, 613.

HUDZIAC, R.M., SCHLESINGER, J. & ULLRICH, A. (1987). Increased expression of the putative growth factor receptor $p185^{HER2}$ causes transformation and tumorigenesis of NIH 3T3 cells. *Proc. Natl Acad. Sci. USA*, **84**, 7159.

HU-LI, J., SHEVACH, E.M., MIZUGUCHI, J., OHARA, J., MOSMANN, T. & PAUL, W.E. (1987). B cell stimulatory factor-1 (interleukin 4) is a potent costimulant for normal resting T lymphocytes. *J. Exp. Med.*, **165**, 157.

LAND, H., PARADA, L.F. & WEINBERG, R.A. (1983). Tumorigenic conversion of primary embryo fibroblasts requires at least two cooperating oncogenes. *Nature*, **304**, 596.

LARCHÉ, M., LAMB, J.R., O'HEHIR, R.E. and 5 others (1988*a*). Functional evidence for a monoclonal antibody that binds to the human interleukin 4 receptor. *Immunology*, **65**, 617.

LARCHÉ, M., LAMB, J.R. & RITTER, M.A. (1988*b*). A novel T-lymphocyte molecule that may function in the induction of self-tolerance and MHC-restriction within the human thymic microenvironment. *Immunology*, **64**, 101.

LARSON, S.M., CARRASQUILO, J.A., KROHN, K.A. and 8 others (1983). Localisation of 131-I-labelled P97-specific Fab fragments in human melanoma as a basis for radiotherapy. *J. Clin. Invest.*, **72**, 2101.

LEE, F.K., YOKOTA, Y., OTSUKA, T. and 9 others (1986). Isolation and characterisation of a mouse cDNA clone that expresses B cell stimulatory factor-1 activity and T cell and mast cell stimulating activities. *Proc. Natl Acad. Sci. USA*, **83**, 2061.

MACH, J.P., BUCHEGGER, F., FORNI, M. and 7 others (1981). Use of radiolabelled monoclonal anti-CEA antibodies for the detection of human carcinomas by external photoscanning and tomoscintigraphy. *Immunol. Today*, **2**, 239.

MERLINO, G.T., XU, Y.H., ISHII, S. and 5 others (1984). Amplification and enhanced expression of the epidermal growth factor receptor gene in A431 human carcinoma cells. *Science*, **224**, 417.

MILLER, R.A., OSEROFF, A.R., STRATTE, P.T. & LEVY, R. (1983). Monoclonal antibody therapeutic trials in seven patients with T cell lymphoma. *Blood*, **62**, 988.

MOSMANN, T.R., BOND, M.W., COFFMAN, R.L., OHARA, J. & PAUL, W.E. (1986). T cell and mast cell lines respond to B cell stimulatory factor-1. *Proc. Natl Acad. Sci. USA*. **83**, 5654.

PARK, L.S., FRIEND, D., SASSENFELD, H.M. & URDAL, D.L. (1987). Characterisation of the human B cell stimulatory factor-1 receptor. *J. Exp. Med.*, **166**, 476.

SLAMON, D.J., CLARKE, G.M., WONG, S.G., LEVIN, W.J., ULLRICH, A. & McGUIRE, W.L. (1987). Human breast cancer: correlation of relapse and survival with amplification of the HER-2/neu oncogene. *Science*, **235**, 177.

SPITLER, L.E., RIO, M.D., KHENTIGAN, A. and 12 others (1987). Therapy of patients with malignant melanoma using a monoclonal anti-melanoma antibody-ricin A chain immunotoxin. *Cancer Res.*, **47**, 1717.

ULLRICH, A., COUSSENS, L., HAYFLICK, J.S. and 12 others (1984). Human epidermal growth factor receptor cDNA sequence and aberrant expression in A431 epidermoid carcinoma cells. *Nature*, **309**, 418.

WILLCOX, H.N.A., SCHLUEP, M., RITTER, M.A., SCHUURMAN, H.J., NEWSOM-DAVIS, J. & CHRISTENSSON, B. (1987). Myasthenic and nonmyasthenic thymoma: an expansion of a minor cortical epithelial subset? *Am. J. Pathol.*, **127**, 447.